# Cognitive and Linguistic Skills Associated With Cross-Linguistic Transfer in the Production of Oral Narratives in English as a Foreign Language by Arabic- and Hebrew-Speaking Children: Finding Common Denominators

**DOI:** 10.3389/fpsyg.2021.664152

**Published:** 2021-08-09

**Authors:** Susie Russak, Elena Zaretsky

**Affiliations:** ^1^English Teacher Training Track, Faculty of Education, Beit Berl College, Kfar Saba, Israel; ^2^Department of Psychology, Clark University, Worcester, MA, United States

**Keywords:** cross-linguistic influence, typological distance, oral narratives, morphosyntactic structures, Semitic languages, English as a foreign language

## Abstract

Many studies have examined literacy and related skills among learners of English as a foreign language (EFL), but little attention has been given to the role of oral language within a cross-linguistic framework despite the fact that English is the most widely spoken additional language today. Oral narratives rely on lexical, morphosyntactic, and conceptual knowledge. An in-depth examination of this modality can shed light on specific associations between cognitive and linguistic L1 and EFL skills and suggest possible mediating variables that assist multilingual speakers in producing complete oral narratives in EFL. The present study examined L1 and EFL contributors to EFL oral narratives produced by native Arabic (*n* = 85) and Hebrew (*n* = 86) speaking sixth graders seeking to identify cross-linguistic influences. We assessed general cognitive skills, phonological memory (PM), lexical, morphosyntactic knowledge, and reading comprehension in L1 (Hebrew speakers), Modern Standard Arabic (MSA, L2), L3 Hebrew (for Arabic speakers) and EFL. The “Cookie Theft” task assessed EFL elicited narratives using modified narrative analysis scales to account for microstructure (lexical and morphosyntactic complexity) and macrostructure (understanding story elements), generating a Total Narrative score. Our results yielded different patterns of underlying psycholinguistic profiles, and cross and within language associations for each group. Strong interactions between L1, L2/L3, and EFL morphological awareness and reading comprehension suggested cross-linguistic transfer. Regression analysis identified the most influential skills supporting EFL narratives for each linguistic group: English reading comprehension (ERC) was essential for Hebrew speakers and English morphological awareness (EMA) for Arabic ones. These results suggested different allocations of cognitive and linguistic resources in EFL narratives. The results also allowed to identify a common mediating skill for both groups. Findings are discussed within the theoretical framework of the *Interdependence Hypothesis*, the *Linguistic Proximity Model*, as well as accounts of direct and indirect transfer, which illuminate the impact of typological distance, general language proficiency and components of linguistic knowledge on cross-linguistic transfer in EFL oral language production.

## Introduction

In today's world, approximately half of the population is multilingual (Grosjean, 2010), and many children acquire literacy in school in a language that they do not speak at home (Nag et al., 2019). Hence, the need to understand the role of crosslinguistic influence (CLI) in both oral and written language domains has become even more relevant. Within this framework, the nature of cross linguistic influences has been attributed to the typological proximity among the languages, the linguistic repertoire of the learners: how many languages they know (Cenoz, 2013), and the levels of proficiency within each language (*Interdependence Hypothesis*, Cummins, 1979, 2014). While CLIs in the domain of written language have been researched extensively, including crosslinguistic transfer of phonological and morphological skills (Schwartz et al., 2007; Saiegh-Haddad and Geva, 2008; Luo et al., 2014), the domain of oral language remains the “Cinderella” of crosslinguistic enquiries. Yet examining oral language skills can provide a window to the repertoire of resources of multilingual learners (Boerma et al., 2016). Oral narrative skills are reliant on underlying cognitive abilities, such as working memory (Kormos and Trebits, 2011), in addition to strong lexical and syntactic knowledge (Dickinson et al., 2019), and activation of metacognitive skills (Cortazzi and Jin, 2007; Kupersmitt et al., 2014). Moreover, they comprise a rich source of information regarding language development (Gagarina et al., 2015). In light of the prevalence of multilingualism in today's society, an examination of cross linguistic influence in the oral language domain among typologically distant languages is warranted.

The present study examined English as a foreign language (EFL) oral narratives skill among sixth grade native Arabic and Hebrew speaking children. The typological distance between English, a Germanic language, and Hebrew and Arabic, both Semitic languages, is obvious. However, while Arabic and Hebrew share typological characteristics, Arabic is unique in that it is a diglossic language (Leikin et al., 2014; Saiegh-Haddad and Henkin-Roitfarb, 2014). This means that Arabic speaking children, while speaking a vernacular of Arabic at home, are exposed to the Modern Standard form when they begin school as their second spoken and first written language. They then begin to study Hebrew as their second written and third spoken language in the second grade, and in third grade, they begin to study English as their third written language and fourth spoken language. Thus, we were interested in exploring the cross linguistic impact of this specific multilingual setting on EFL oral language production, particularly in light of the fact that all children in Israel study English as their first foreign language according to the same national curriculum. Of additional importance, we were interested in exploring the possible mediating factors that may support EFL oral language skills in this sample of speakers of two different Semitic languages.

### Cross Linguistic Influences of Cognitive and Linguistic Skills on Oral Language Production

The term “crosslinguistic influence” describes cognitive and linguistic processes that allow to apply knowledge of one language to another language (Moattarian, 2013). Cross linguistic influences in language production among speakers of multiple languages have been attributed to learner characteristics, including number of languages spoken and linguistic proficiency. Cummins' *Interdependence Hypothesis* in bilingualism, for example, claims that second language (L2) skills are dependent in part on L1 language abilities, and that language skills will transfer from L1 to L2 given sufficient exposure and level of proficiency in L2. Thus, well-developed skills in L1 should support the development of similar skills in L2 (Cummins, 1979; Verhoeven, 1994). Within a broader framework of third language acquisition, it has been suggested that third language learners have a larger linguistic repertoire than second language learners, have stronger metalinguistic skills (Jessner, 2008; Huang, 2018), are more experienced with language acquisition processes, and thus, may have developed a unique set of language learning strategies that they can access (Jessner, 2008; Cenoz, 2013). Moreover, as a result of their wider linguistic repertoire, multilinguals may activate both direct routes and indirect routes to additional language learning (Hirosh and Degani, 2018). According to Hirosh and Degani (2018), direct routes include transfer of linguistic skills and knowledge, whereas indirect routes represent cognitive factors such as metalinguistic awareness and working memory.

In addition to learner attributes, cross linguistic relationships are also affected by properties of the languages in contact, such as general typological proximity or more specifically structural similarities. According to the *Linguistic Proximity Model* (Westergaard et al., 2017), structural similarities among the languages of a multilingual learner may lead to facilitative cross linguistic influences, particularly when the learner is aware of fine-grained variations across the grammars of their language repertoire. Non-facilitative influences may occur when the learner does not have a solid grasp of particular linguistic input in the target language and erroneously attributes shared properties between the target language and any of the already learned languages. Within this model, the specific patterns of influence will be determined by the areas of cross language overlap (Westergaard et al., 2017). However, recent research indicates that there is a bidirectional transfer of skills from L1 to L2 (*forward transfer)* and from L2 to L1 (*reverse* transfer) (Jarvis and Pavlenko, 2008; Kim and Piper, 2019). Pavlenko and Jarvis (2002) examined bidirectional influences of L1 (Russian) and L2 (American English) oral production and found that not only did Russian influence English, but there was also a direct influence of English (L2) on L1 (Russian) oral production. Kim and Piper (2019) found bidirectional influences between Kiswahili and English (official languages in Kenya) literacy skills. The present study examines cross linguistic influences in EFL oral narrative production among monolingual Hebrew speakers and multilingual Arabic speakers.

### Oral Narratives

Oral narratives have long been recognized as a valid measure of linguistic growth in monolingual and bilingual children from different linguistic backgrounds (Berman and Slobin, 1994; Pavlenko, 2002; Soodla and Kikas, 2010). The interest in narratives is fueled by different disciplines: linguistics, sociology, cognitive psychology (Iluz-Cohen and Walters, 2012). Producing a narrative requires the integration of different cognitive and linguistic skills, e.g., interaction of lexical, morphosyntactic, and general discourse knowledge (Boerma et al., 2016), along with metacognitive skills (Cortazzi and Jin, 2007; Kupersmitt et al., 2014).

Growing narrative abilities also coincide with the development of executive functions, in this instance the ability to plan, i.e., organizing the story in sequential order (Friend and Bates, 2014), making narratives a perfect vehicle to access linguistic and cognitive growth in children. Expressing one's own and others' perspectives through lexical diversity and application of appropriate morphosyntactic structures (Moonsamy et al., 2009) involves both production and comprehension of multiple utterances and represents the current level of linguistic and conceptual knowledge of an individual (Justice et al., 2010). For these reasons, narratives have been examined from the point of view of (1) global characteristics, i.e., macrostructure, or producing a narrative based on the understanding of the thematic orientation, as it accounts for the “mental representation of events” (Berman, 1995, p. 287), and (2) overall grammatical complexity, e.g., microstructure, which includes lexical diversity as well as morphosyntactic knowledge, to represent the meaningful use of grammatical structures to allow listener's understanding (Justice et al., 2010). Moreover, strong correlations were found between these two structures of narratives, indicating that better lexical and morphosyntactic knowledge results in better global representation of narrative (Terry et al., 2013). Research also suggests that there is a strongly implied interaction between lexical and grammatical knowledge across different languages, such as Italian, Hebrew, Icelandic, etc. (Thordardottir et al., 2002). Moreover, Thordardottir et al. (2002) suggested that this interaction is due to a single mechanism which supports the development of both lexical and grammatical knowledge. Speakers of multiple languages also showed interaction between lexical and grammatical knowledge, when they produce narratives in their non-native languages (Marchman et al., 2004; Simon-Cereijido and Gutiérrez-Clellen, 2009; Gagarina et al., 2015; Gagarina, 2016). However, there is no consensus regarding which particular factors, e.g., lexical, morphosyntactic, or general cognitive skills, may play mediating roles among learners of foreign languages in general, and among learners of EFL specifically, in their attempts to produce cohesive oral narratives in English. Despite the proposed interaction between lexical and morphological knowledge, there is also evidence that lexical and morphological knowledge may separate among second language learners, depending on the morphological tasks' reliance on general vocabulary (Shahar Yames et al., 2018).

Since the time when Labov (1972) delineated structural and functional aspects of narratives as macro- and micro-structure, or cohesion and coherence, researchers have consistently used specific markers to account for the completeness of narratives. The macrostructure assessment includes story setting (time and place), identifying the protagonist, references (use of pronouns with antecedents), and attempts to solve the problem (Pearson, 2002; Uccelli and Păez, 2007; Heilman et al., 2010). At the same time, when producing an oral text, a narrator in EFL must also show the ability to use appropriate lexical and morphosyntactic structures (microstructure, or coherence) in the non-native language in order for the narrative to be understood by the listener (Iluz-Cohen and Walters, 2012). This includes extensive vocabulary, appropriate knowledge of morphological inflections, as well as sentence complexity, e.g., use of cohesive devises, such as conjunctions. While research showed that the stories produced by multilingual speakers in their L1 and L2 may not really differ in the macrostructure, there were substantial differences in the appropriate use of the linguistic elements in L2 production (Pearson, 2002). Moreover, neither Pearson (2002), nor Uccelli and Păez (2007) later found any cross-linguistic transfer in children's narratives. On the other hand, Castilla et al. (2009) did find correlations across L1 and L2 (Spanish and English in this instance) in morphosyntactic knowledge, which indicated that there are some aspects of linguistic knowledge that actually can be transferred cross-linguistically. More recent research with Cantonese-English speaking preschoolers showed that Cantonese micro- and macrostructure predicted English micro- and macrostructure, again suggesting cross-linguistic transfer, as well as bidirectional influences (Rezzonico et al., 2016). This particular study is important, as it examined cross-linguistic transfer among typologically different languages. There is also substantial evidence that the transfer can occur on the level of morphosyntax, e.g., development of relative clauses in Cantonese-English bilinguals (Yip and Matthews, 2007), as well as in morphological awareness. For example, Pasquarella et al. (2011) showed bidirectional transfer of morphological awareness between English compound morphology and Chinese vocabulary acquisition, as well as English compound morphology predicting Chinese reading comprehension. However, the abovementioned studies examined written language. Nevertheless, based on these results, we may infer which L1 skills might contribute to producing L2 narratives among bilingual individuals, and individuals who are learning EFL with typologically different L1s. In other words, it is possible to postulate that there is a mediating effect of specific L1 skill that will account for cross-linguistic transfer even in the oral language domain.

### Language Typology: Structural Similarities and Differences Between Arabic/Hebrew and English

Arabic and Hebrew are Semitic languages. As such, they are both morphologically rich with words being formed by combining roots comprised of two to four letters with patterns or templates: these are usually inserted between the root letters in order to create words with meaning. Some words are also formed by affixation at the beginning or end of the word. Verbs are inflected for number, gender, person (first, second, and third), and tense, or aspect to create three tenses: past, present, and future. Nouns are inflected for number and gender. Pronominal suffixes can be attached to verbs and nouns, thereby adding an additional layer of morphological complexity to words, so that most words are multi-morphemic. Research indicates that as a result of the morphological complexity of the language, Arabic and Hebrew speaking children show signs of morphological sensitivity at an early age and are well able to attend to internal word structure (Ravid, 2001; Gillis and Ravid, 2006; Saiegh-Haddad and Taha, 2017; El Akiki and Content, 2020). Moreover, there is empirical evidence that young bilingual Hebrew and Arabic speakers outperform monolinguals on tasks of derivational morphology, as a sign of positive cross linguistic influences (Asli-Badarneh and Leikin, 2019). These findings suggest that cross-linguistic influences can be traced among languages that belong to the same typological group, such as Hebrew and Arabic (Schwartz et al., 2016; Asli-Badarneh and Leikin, 2019). It has been further suggested that since Arabic morphology is more complex than Hebrew morphology, Arabic speaking bilinguals may have an advantage when they transfer skills from their more complex L1 morphological system to the less complex L2 Hebrew system (Chen and Schwartz, 2018). However, despite shared linguistic and structural features between Arabic and Hebrew, Arabic is unique in that it is a diglossic language, characterized by linguistic distance between the spoken dialects and the Modern Standard form (Saiegh-Haddad, 2003). Moreover, there may be great variance across spoken dialects in the areas of phonology, morphology, and semantics (Saiegh-Haddad and Henkin-Roitfarb, 2014), while the written form is stable. Children begin to learn the Modern Standard form formally when they enter school, although they are exposed to it informally in their environment from an early age though literature and media. Thus, Arabic speakers study academic subjects in school in Modern Standard Arabic (MSA), a language that is not their home language (spoken dialect). As a result of their exposure to two forms of the language from an early age, some researchers suggest that Arabic speaking children are, in essence, can be considered bilingual (Eviatar and Ibrahim, 2000), e.g., the spoken form is their L1 and the Modern Standard form is their L2, even before they begin to study Hebrew. Studies have also indicated that the linguistic distance between the two forms of Arabic language may result in difficulties with the acquisition of the Modern Standard form, and other literacy related skills (Saiegh-Haddad, 2003, 2007). Moreover, these difficulties persist beyond the initial years of literacy acquisition (Abdelhadi et al., 2011).

In contrast to Semitic languages, English is a Western Germanic language with a relatively simple morphological structure. Morphemes are affixed onto base words producing multimorphemic words, which can be inflectional or derivational in nature. Inflectional morphemes change tense or number of free morphemes, whereas derivational morphology can change the part of speech or the meaning of the base word. In addition to the three basic tenses (past, present, future), English verb tenses are complicated by the use of aspectual forms, which contribute an additional level of linguistic complexity. And yet, English has been described as a morphologically impoverished language due to the fact that, in comparison with Semitic languages, there are hardly any markings of agreement for gender, number, or person (DeKeyser et al., 2010; Tsarfaty and Sima'an, 2010).

In addition, word order within a sentence in Semitic languages, is rather flexible. This is because syntactic characteristics are determined based on morphological information embedded within words, above and beyond word order within a sentence (Tsarfaty and Sima'an, 2010). Thus, while the dominant word order for Hebrew sentences is SVO, and VSO for Arabic sentences, variability in word order appears and is accepted in both languages (Schwartzwald, 2011). Moreover, verbless constructions exist in both languages. In contrast, English word order within sentences is relatively fixed according to the SVO pattern so that grammatical patterns are reliant on syntax along with meaning. The present study examines oral language skills in EFL among Arabic and Hebrew speaking pupils in sixth grade, thus, these particular differences between Hebrew, Arabic and English language structure provide an important frame of reference.

### Importance of English and EFL Learning Policy in Israel

English is a global language (Crystal, 2012). It is also the most widely taught foreign language today. In Israel, English holds a unique status. It is a semi-official language and the first foreign language studied in all schools. It is the key to international economic growth, and social communication, as well as the gatekeeper to academic advancement. It is also a compulsory subject for the high school matriculation certificate. Moreover, English proficiency is required as a prerequisite for entrance into higher education.

Based on the language learning policy of the country, Arabic speakers are taught in school in Arabic, and Hebrew speakers are taught in Hebrew. Arabic speaking pupils must also learn Hebrew as their second written and third spoken language, beginning in the second grade or even earlier (Amara, 2018). Hebrew speaking pupils only begin to study Arabic in the 7^th^ grade, if at all. Formal instruction in English as the first foreign language (EFL) for both populations begins in 3^rd^ grade.^1^ Thus, while Hebrew speakers study EFL as their second language, native Arabic speakers study EFL as the 3^rd^ written language and fourth spoken one. Moreover, the status of each of the languages is evident from their appearance in the linguistic landscapes of the community and the school settings. Among the Hebrew speaking communities, Hebrew is dominant in the linguistic landscape, followed by English. In most Arabic speaking communities, Arabic is the dominant language in the linguistic landscape followed by Hebrew. English is hardly present, except for the region of East Jerusalem where it is the second most prominent language after Arabic (Amara, 2018). A similar situation exists within the school linguistic landscapes, at least in the Arabic speaking population, where Arabic is the most prominent language followed by Hebrew with little or no representation of English (Amara, 2018).

Based on the national guidelines, all pupils in Israel learn EFL according to the same English curriculum and teaching materials, regardless of their L1 backgrounds. The first year of study (3^rd^ grade) is dedicated to building basic oral skills and introducing the letters of the alphabet. Pupils in 3^rd^ grade study English for two 45-min lessons a week. From 4^th^–6^th^ grades pupils receive four 45-min EFL lessons a week. In the 4^th^ grade, pupils continue to build their lexical knowledge while beginning to learn how to read. By the last year of elementary school (6^th^ grade), pupils are expected to reach a basic level of oral and written language proficiency.

In reality, however, after the first year of oral language instruction, there is little, if any time specifically devoted to the further explicit development of authentic oral language skills (Al Hosni, 2014). While the revised elementary English curriculum^1^ relates to oral language skills, the expectation is that the pupils will be able to manage short, rehearsed utterances, and not necessarily produce spontaneous language. Moreover, the reality of large numbers of pupils in each class does not really allow for individual practice or use of oral language skills in school. Additionally, while the revised curriculum highlights the importance of English oral skills in the classroom, many teachers prefer to use their L1 (Orland-Barak and Yinon, 2005; Timor, 2012), which further limits exposure to spoken English.

### Present Study

The present study explores the contribution of cross linguistic influence to oral narrative production in EFL among a sample of Hebrew and Arabic speakers. To our knowledge, this is the first study that directly examines cross language influences and possible specific influentual factors between Semitic L1 and EFL through oral narratives. Previous studies concerned with narrative production among bilingual individuals compared macro- and micro-structures in relation to the differences of the output between L1 and L2 of participants (Uccelli and Păez, 2007; Gagarina, 2016; Lucero, 2018). However, there was no specific data relating to which L1 skill(s) had a mediating effect on L2 narratives, as well as the possibility that different EFL skills may prove to be more influential for oral EFL production among Arabic and Hebrew speakers. The present study addresses this gap by examining oral narratives produced by 6^th^ grade speakers of Arabic and Hebrew in their 4th year of learning EFL, within a cross-linguistic framework, in search for possible mediators between Semitic languages and English. For these purposes, we did not compare L1 and EFL narratives, but rather examined the proficiency of the participants in linguistic and literacy domains in L1 Hebrew and L2 Modern Standard form of Arabic (MSA) and EFL, as well as general cognitive skills, as possible contributing factors to the quality of EFL oral narratives.

Our research questions were as follows:

1. What is the contribution of L1 Hebrew linguistic skills (morphology, reading comprehension), as well as phonological memory (PM) (word repetition), and general cognitive abilities, to micro- and macro-structures in EFL oral narratives among monolingual Hebrew speakers?
  We hypothesized that in line with the *Interdependence hypothesis* (Cummins, 1979, 2014) we should see direct cross-linguistic influence with strong L1 skills supporting similar skills in L2. However, if non-facilitative influences are observed, these findings may be attributed to either typological differences between Hebrew, a Semitic language, and EFL, as postulated by the *Linguistic Proximity model*, or low levels of EFL proficiency.
2. What is the contribution of MSA and Hebrew (L3) linguistic (morphology, reading comprehension), as well as cognitive skills (phonological and general cognitive abilities), to micro- and macro-structures in EFL oral narratives among multilingual Arabic speakers?
  We hypothesized that since the Arabic speakers in the present study are multilingual and have a broader linguistic repertoire than the monolingual Hebrew speakers, there may be a different allocation of cognitive and linguistic resources that will impact the nature of the cross linguistic influences on EFL narrative skills. Moreover, there may be both direct transfer of linguistic skills and knowledge between the same Arabic and Hebrew skills, as well as indirect transfer of cognitive and metalingustic skills to EFL narratives, as long as their Arabic and Hebrew linguistic skills are at a sufficient level of proficiency to support transfer.
3. What is the contribution of EFL linguistic skills [English reading comprehension (ERC), English morphological awareness (EMA)] to micro- and macro-structures in EFL oral narratives among monolingual Hebrew speakers and multilingual Arabic speakers?
  We hypothesized that as macrostructure is a measure of the overall ability to represent global characteristics of a narrative, ERC scores, representing an understanding of the story events, would have a positive relationship with EFL macro- and Total narrative scores. We further hypothesized that since microstructure is a measure of lexical diversity as well as morphosyntactic knowledge (Justice et al., 2010), EMA scores would have a positive relationship with EFL micro- and Total narrative scores. In the event that the abovementioned EFL linguistic skills show a positive relationship with all EFL narrative measures, it will be possible to infer that these skills may play a modulating role in the relationship between Arabic, Hebrew, and EFL narratives.
4. Are there L1(Hebrew)/L2 (MSA)/L3 (Hebrew for Arabic speakers) skills that may have cross linguistic influences in the relationship between EFL skills and EFL oral narratives? We hypothesized that possible cross linguistic relationships between measures of EFL (ERC and EMA) and L1 Hebrew/L2 MSA/L3 (Hebrew for Arabic speakers) MA and RC, as well as EFL total narrative structure should indicate an indirect mediation effect on the quality of the EFL narrative.

## Methods

### Participants

The participant pool was comprised of two linguistic groups: 86 native mono-literate sixth grade Hebrew speaking pupils for whom English is the second language being learned in school (43 females), and 85 native 6^th^ grade Arabic speaking pupils, who learn spoken language as L1, MSA as L2, learn Hebrew as L3 and then begin learning EFL. Therefore, this group can be considered “multilingual,” as English is third written language and the fourth spoken language being learned in school (44 females). All of the participants were between the ages of 10 and 11 at the time of data collection. The Arabic speaking pupils were chosen from four different schools, and the Hebrew speaking pupils were chosen from six different schools in the central area of Israel. As the Arabic speaking participants were from several different cities, their spoken dialects varied in relation to the city where the participants lived. The average socio-economic index for the Arabic speaking school was 5.66, and for the Hebrew speaking schools 2.95 on a scale of 1–10 where 1 is the highest and 10 is the lowest. However, very large discrepancies can be found within the Arabic speaking populations that were included in the study. As a case in point, in our groups, there were significant individual differences on task results, as can be seen in the very large standard deviations in Table 1^2^. The protocol of this study was approved by the Ministry of Education Chief Scientist Bureau. Pupils chose to participate on a voluntary basis and all parents signed a consent form. Pupils with learning disabilities or pupils who are fluent in languages in addition to Hebrew or Arabic were excluded from the sample.

**Table 1:** Descriptive statistics in percentage scores (except for Arabic morphological word derivation task which are presented as raw scores).

**Task**	**Hebrew speakers**	**Arabic speakers**
	***Mean (SD)***	***Mean (SD)***
	***N* = 86**	***N* = 85**
Microstructure	17.64 (15.18)	12.16 (12.79)
Macrostructure	47.83 (20.15)	36.86 (19.40)
Total narrative	26.47 (15.56)	19.38 (13.79)
Raven	87.58 (11.5)	79.89 (13.06)
English word repetition (as a measure of phonological memory)	91.26 (9.83)	93.08 (8.24)
Arabic morphological word derivation^a^	X	15.32 (5.14)
Arabic morphological root pattern awareness	X	89.23 (15.69)
Arabic reading comprehension	X	76.34 (23.31)
Hebrew morphological real word derivation	83.59 (14.23)	16.93 (12.96)
Hebrew morphological pseudo word derivation	77.23 (14.77)	26.61 (22.07)
Hebrew reading comprehension	80.50 (17.19)	X
English morphological awareness	65.36 (21.44)	44.97 (29.77)
English reading comprehension	61.01 (21.44)	43.37 (26.19)

a *This score is reported as a raw score due to the nature of the task*.

### Tasks

Language tasks in Arabic and Hebrew were adapted from existing standardized tools (Arabic—Asadi et al., 2015; Hebrew—Shany et al., 2006). All the Arabic tasks were administered in the MSA (which, as mentioned earlier, is considered the first written language that the Arabic speakers acquire but the second spoken language). Arabic speakers were also tested in Hebrew—their second written and third spoken language. While most of the Arabic and Hebrew tests have norms, some of the tasks were shortened for the purposes of the present study and therefore the norms can only be used to give an indication of performance trends. Further, the Hebrew tasks were not designed to be administered to non-native Hebrew-speaking pupils, so the scores for the Arabic speakers cannot be evaluated with relation to the Hebrew norms. All tests, in both languages, were administered by native speakers of the language of the participants. In what follows, the tasks will be described, according to language of administration.

#### Raven Matrices (As a Measure of General Cognitive Ability)

Raven Colored Matrices (Raven et al., 1976), as a test of non-verbal intelligence, is comprised of three sets of tasks with 12 items in each set (maximum possible score = 36). The participant is required to select the correct pattern to complete a matrix out of six options. Prior to testing, participants received an explanation as to how to fill out the answer sheet. This task was administered in Arabic for the Arabic speakers and Hebrew for the Hebrew speakers (Cronbach's Alpha 0.87).

#### Arabic Assessment Battery

##### Reading Comprehension (Asadi et al., 2015)

This task requires the participants to read a short passage (205 words in length) and answer nine multiple choice questions. The expository text is about a subject that is relevant to children in the sixth grade (Cronbach's alpha 0.71).

##### Morphological Choice (Asadi et al., 2015)

This task examines students' awareness of the roots in words using an *odd one out* format. The pupil is presented with four written words in a row and is required to choose the item that does not belong based on a change to the root. Roots are changed in the words by switching the order of the first or second letter. There are a total of 20 sets of four items (Cronbach's alpha 0.90).

##### Morphological Word Derivation (From Roots) (Asadi et al., 2015)

In this task, the pupil is presented with five three-letter roots and has 1 min to derive as many words from each root as possible. One point is given for each correctly derived word (Cronbach's alpha 0.82).

Arabic speakers were also assessed on Hebrew MA (real and pseudo word derivations), as they learn Hebrew as their second written language (see descriptions of the tasks below).

#### Hebrew Assessment Battery

##### Reading Comprehension

Participants were presented with a reading passage (218 words) which was adapted from a sixth grade Hebrew textbook. The expository text is about a subject that is relevant to children in the sixth grade. There were nine multiple choice questions. Prior to administration, four Hebrew language teachers rated the text as grade appropriate (Cronbach's alpha 0.33).

##### Morphological Real Word Derivation (Shany et al., 2006)

This task comprised nine sentences where one word was missing from each sentence (*cloze* procedure). The pupils were given a three-letter root pattern and were required to fill in the missing word in the sentence by deriving the correct form of the word in the context of the given sentence (Cronbach's alpha 0.91).

##### Morphological Pseudo Word Derivation (Shany et al., 2006)

This task comprised 12 sentences where one word was missing from each sentence (*cloze* procedure). The pupils were given a three-letter pseudo root pattern and were required to fill in the missing pseudo word in the sentence by deriving the correct form of a word in the context of the given sentence. Success on this task was dependent on an understanding of the morphological patterns governing word construction in Hebrew. There were 12 items in this task (Cronbach's alpha 0.89).

#### English Assessment Battery

##### Word Repetition (As a Measure of Phonological Memory)

Pupils heard a word and were asked to repeat the word after the tester. Twenty-five items for this task were chosen based on number of syllables (2–4) and level of familiarity (None of the chosen words appear in the first 1,200 words from the list of lexical items of the English Inspectorate of the Ministry of Education, Israel. Thus, as the chosen words were unfamiliar to the participants, they were, in essence, pseudo words). This task tapped into phonological short-term memory (Cronbach's alpha 0.66).

##### Reading Comprehension

This task included a passage (101 words), composed of vocabulary taken from the list of the first 1,200 words proposed by the English Inspectorate of the Ministry of Education, Israel (State of Israel, Ministry of Education Pedagogical Secretariat, Language Department, Inspectorate for English Language Education, 2020), followed by seven multiple choice questions. The topic was chosen by reviewing the content of the textbooks used in the fifth and sixth grades. Once the text and questions were written, the passage was given to three different elementary school English teachers who were asked to judge if the passage and questions were appropriate for the sixth graders. Each of the teachers assessed the passage as suitable. This task also served as a proxy for EFL vocabulary task, because in order to understand a written text, one must have a substantial knowledge and comprehension of vocabulary items (Cromley and Azevedo, 2007) (Cronbach's alpha 0.73).

##### Morphological Awareness

This task comprised nine sentences where one word was missing from each sentence (*cloze* procedure). The pupils were given a base word and were required to fill in the missing word in the sentence by deriving the correct form of the base word in the context of the given sentence. Morphological structures targeted included both inflectional and derivational morphemes (for example: I teach English to my pupils. I am a ____.) (Cronbach's alpha 0.77).

#### Oral Narrative Task in English (Dependent Variable)

All participants were asked to produce an elicited oral narrative in EFL based on the “Cookie Theft” task (Goodglass and Kaplan, 1983). This particular task presents extensive opportunity for children to describe the salient elements (e.g., mother washing the dishes, water overflowing from the tap, a boy, attempting to get cookies from a jar stored in a cupboard, the chair that is falling down, the girl stretching her hand to reach the cookies) as well as background features (e.g., trees, grass, clothes, etc.) of a black and white picture. All children had the prompt “Tell me about this picture” to orient them to all aspects of the picture, however, it is up to the narrator to relate to specific details. Despite its static nature, the picture presents the opportunity to use a variety of lexical items, including abstract and concrete words, and verbs in appropriate tenses, to construct grammatically correct sentences, and provide cohesive references (Cummings, 2019). Moreover, evidence suggests that narratives produced based on static picture show higher mastery than the ones produced on a series of pictures (e.g., famous “Frog Where Are You” by Mercer Myer) (Cornaglia et al., 2017).

The narratives were recorded and then transcribed by a trained research assistant and rechecked by the second author. The data was then analyzed using the items included in Narrative Assessment Protocol (short form, Justice et al., 2010) to assess the microstructure of the narratives. The following items from the NAP short form were included to assess microstructure: number of complete sentences, number of complex sentences (e.g., sentences with clauses and conjunctions), instances of noun plural inflection, verb morphology, such as present progressive (e.g., auxiliary + main verb), copula *be*, 3^rd^ person singular, past tense. The macrostructure analysis, i.e., completeness of the narratives, was adapted from Narrative scoring scheme (Heilman et al., 2010). In what follows the method of assessment will be described, first for micro- and then for macro-structure.

The microstructure analysis was comprised of two indices representing cohesion: (1) The “*Complete Sentences”* index which was a combination of all the required linguistic elements in their correct forms (i.e., use of appropriate inflectional verb morphology and maintenance of the English word order); and (2) the “*Conjunction Cohesion”* index, which was a measure of sentence complexity, e.g., use of clausal structures. Each instance of full sentence, clausal structure and correct use of inflectional morphology received a score of 1. The maximum total possible score (sum of “*Complete Sentences”* and “*Conjunction Cohesion”*) for the microstructure was 36, similar to the one suggested by the NAP short form, where total instances of each of the correctly produced required elements could not be assigned a score higher than 3 (the short form uses 3+ for the highest possible frequency of use) (Cronbach α = 0.57). This arrangement was deemed appropriate for the purposes of this study, as many students produced only labels for the items they saw in the picture. However, many nouns used by participants were inflected for plurality, allowing the student to receive a score.

To assess the macrostructure, we modified the narrative scoring scheme suggested by Heilman et al. (2010). Since we used static stimulus, the picture was divided into three episodes: (1) Mother (washing dishes, holding plate, drying dishes, looking out of the window, etc.); (2) boy/girl (reaching for cookies, giving cookies, eating cookies, climbing chair, etc.); and (3) water overflowing from the sink (water on the floor, wet floor, mother/children don't see it, etc.) with five indices each (e.g., Topic maintenance, Event Sequencing, Information, Referencing, Character ID) scored on a 0–3 scale, where 0 signified non-observed ability, resulting in a total of 15 points (Cronbach α = 0.74) The rater agreement (Cohen's kappa, Cohen, 1960) for the transcription and scoring was at 0.87, which is “almost perfect” (82% and above is reliable) (see Appendix 1 for a detailed description).

#### Data Analysis

The present study set out to explore the contribution of cognitive and linguistic skills to the production of oral narratives in EFL, among L1 Hebrew and Arabic speakers in 6^th^ grade, as well as identify the underlying mechanisms that lead to cross-linguistic transfer and the mediating factor(s) that may be associated with this process. We included descriptive statistics of all test results in both linguistic groups, which are represented through means and standard deviations. Interpretations of the scores were done in accordance with norms for tasks that were norm-referenced. The main part of the analysis was based on hierarchical multi-variate linear regression models. Prior to the named analyses assumption of linearity relationship was tested by curve fitting analysis that tested the significance of the linear relationships between the predictors in the models and the outcome variable within each language group. This analysis revealed that these bivariate associations had significant linear relationship with the outcome variable.

The hierarchical modeling strategy was based on the following logic: first we monitored general cognitive abilities, represented in the study by Raven Colored Matrices and by English word repetition (EWR) (cognitive/linguistic task for assessing PM). Then, we wanted to see the additional contribution of language tasks in first and additional languages. For Hebrew speakers, this meant all tasks in Hebrew first and then in EFL. For Arabic speakers, Arabic language measures (MSA) were entered first, followed by language measures in Hebrew (L3) and then in EFL (L4). In terms of the final structure of multi-variate linear regression, there were three models for Hebrew speakers and four models for Arabic speakers. The Durbin-Watson test for independence of errors, showed values for both language groups were between 1.50 and 2.00, which indicates that residuals are uncorrelated.

The investigation of the L1/L2/L3 skills that support EFL oral narratives used the assumptions of the *Confounding hypothesis* (MacKinnon et al., 2000), that a 3^rd^ variable (Z) may explain the relationship between the predictor (X) and outcome variable (Y), by having an impact on both. We include the results of the bootstrapping and Sobel Test procedures, as the statistical analysis for mediation and confounding are similar, except for assumption of the directionality (Hayes, 2019). All analyses were performed using SPSS25, results were considered significant when *p* ≤ 0.05.

## Results

The present study set out to explore the contribution of cognitive and linguistic skills to the production of oral narratives in EFL, among native Hebrew and Arabic speakers in 6^th^ grade, as well as identify the underlying mechanisms that lead to cross-linguistic transfer. We first established base-line Arabic, Hebrew and EFL proficiency scores in order to validate the assumptions of the *Interdependence Hypothesis*, that suggests reliance of additional language acquisition on native language proficiency.

Table 1 represents the percentage scores on all tasks, except for the score on the Arabic morphological derivation, which was reported as a raw score because of the nature of the task, where there was no definite number for the total possible score. The Arabic speakers were tested in both Arabic (MSA) and Hebrew (L3), before being tested in EFL. Appendix 2 presents a list of all the abbreviations for tasks reported in the results and discussion sections.

As can be seen in Table 1, each group showed a unique psycholinguistic profile. While the Arabic reading comprehension (ARC) and morphological choice tasks were shortened versions of the same tasks from the standardized Arabic test battery (Asadi et al., 2015), the scores in Table 1 all fall within the normative range as compared to the scores for the similar full tasks. The scores on the adapted version of the morphological derivation task are also reflective of the normative scores from the standardized Arabic test battery (Asadi et al., 2015). Thus, it is possible to say that the Arabic speakers in the present study exhibited adequate normative proficiency in MSA. With regards to the performance of the Arabic speakers on the Hebrew (L3) tasks, scores among the Arabic speakers were higher on the Hebrew morphological pseudo word derivation task than on the morphological real word derivation task. Scores on the EFL tasks fell within the moderate range of proficiency.

The scores for the Hebrew speakers on the two Hebrew (L1) morphological awareness tasks were within the average high to high range, based on the given norms for those tasks (Shany et al., 2006). While the reading comprehension task was not norm-referenced, a mean score of 80 could be considered within the high average range. Scores for the EFL tasks among the group of Hebrew speakers fell in the average high range of proficiency.

### Research Questions 1, 2, and 3

The first research question addressed the contribution of different cognitive and linguistic skills in Hebrew, as L1 to the ensuing EFL narratives. Our second research question addressed the contributions of cognitive and linguistic skills in Arabic as the first written language of the participants, along with Hebrew as the second written language and third spoken language acquired by Arabic speakers, and micro- and macro-structures of EFL narratives. The third question explored the contribution of EFL linguistic skills (ERC, EMA) to EFL oral narratives among the monolingual Hebrew and multilingual Arabic speakers. In what follows, results will be presented for each language group separately.

#### Hebrew-Speaking Participants

##### Correlation Analysis

In this section, we present only the most significant correlations to identify the variables that were used in *Regression Analysis. Raven*, as a measure of general cognitive abilities did not show correlations with any measure of Hebrew, EFL, and narrative components. However, EWR as a measure of PM, strongly and significantly correlated with every measure of Hebrew assessments (two tasks of HMA and HRC), as well as every component of EFL narratives (see Table 2 for results). Both, HMA_pseudo_ and HMA_real_ tasks strongly and significantly correlated with all elements of EFL narratives, with HMA_pseudo_ being the strongest, and ERC. Moreover, HRC showed significant correlation with ERC. EFL tasks, e.g., EMA and ERC showed strong intra language correlations with each other and with all elements of narrative structure (see Table 2 for all results).

**Table 2:** Hebrew speakers: correlations among all measures and English oral narrative scores.

**Variables**	**1**	**2**	**3**	**4**	**5**	**6**	**7**	**8**	**9**	**10**
1. E Micro	–									
2. E Macro	0.73^**^	–								
3. Total narrative	0.97^**^	0.88^**^	–							
4. Raven	0.01	0.04	0.02	–						
5. HMA_real_	0.22^*^	0.22^*^	0.23^*^	0.04	–					
6. HMA_pseudo_	0.25^*^	0.26^*^	0.27^*^	0.19	0.40^**^	–				
7. H RC	0.20	0.15	0.19	0.13	0.05	0.18	–			
8. E WR	0.27^*^	0.35^**^	0.32^**^	0.16	0.26^*^	0.26^*^	0.27^*^	–		
9. EMA	0.26^*^	0.26^*^	0.28^*^	0.03	−0.06	0.07	0.03	0.07	–	
10. ERC	0.48^**^	0.52^**^	0.53^**^	0.04	0.13	0.35^**^	0.31^**^	0.18	0.47^**^	–

*HMA_real_, Hebrew morphological real word derivation task; HMA_pseudo_, Hebrew morphological pseudo word derivation task; RC, reading comprehension; H, Hebrew; E, English; MA, morphological awareness; WR, word repetition*.

* *p < 0.05;*

** *p < 0.01*.

##### Hierarchical Regression

The total narrative score was used as the dependent measure, since it represents both, the micro- and macro-structures of the narrative. The independent variables were entered in an hierarchical fashion: the first block contained the Raven and EWR scores as representations of general cognitive skills (Model 1). In the second block the Hebrew language variables (both MA tasks and RC) were added (Model 2). In the third block the English language variables were added (Model 3). The final model was significant and explained 35% of the variance in the total narrative score [*F*_(2,75)_ = 10.10, *p* < 0.001]. Two predictors were found to have a significant association with the dependent variable: ERC (β = 0.40, *p* < 0.001) and EWR (β = 0.24, *p* < 0.01). No significant associations were found for the other variables in the model. The regression model for Hebrew is presented in Table 3.

**Table 3:** Hierarchal linear regression analysis for contributors to total narrative score among Hebrew speakers.

	**Model 1**			**Model 2**			**Model 3**		
**Variable**	***B***	***SE B***	**β**	***B***	***SE B***	**β**	***B***	***SE B***	**β**
Raven	−0.11	0.39	−0.03	−0.23	0.40	−0.06	−0.15	0.36	−0.04
English word repetition	0.56	0.17	0.35^***^	0.43	0.18	0.27^**^	0.38	0.16	0.24^**^
Hebrew morphological awareness pseudowords				0.12	0.13	0.11	−0.01	0.12	−0.01
Hebrew morphological awareness real words				0.14	0.12	0.13	0.15	0.11	0.14
Hebrew reading comprehension				0.12	0.11	0.12	0.03	0.10	0.04
E morphological awareness							0.07	0.08	0.09
E reading comprehension							0.21	0.06	0.40^***^
*R^2^*		0.12			0.17			0.35	
*R^2^* change		0.12			0.05			0.18	
*F* for change in *R^2^*				*F*_(3,77)_ = 1.58 *p* = 0.20			*F*_(2,75)_ = 10.10, *p* = 0.000		

** *p is significant at 0.01;*

*** *p is significant at 0.001*.

#### Arabic-Speaking Participants

##### Correlation Analysis

Notable differences were observed in the relationships between the same cognitive and linguistic variables among Arabic-speaking children. As Arabic speakers learn Hebrew as their second written and third spoken language, Hebrew measures were included in the analysis, specifically to identify possible relations between Arabic and Hebrew morphological knowledge as part of the cross linguistic language profile. Firstly, *Raven*, as a measure of general cognitive skills, did not correlate with any of Arabic morphological awareness tasks (AMA_choice_ and AMA_deriv_), but significantly correlated with ARC, HMA_real_, and all aspects of EFL narratives (Table 4). EWR, as a measure of PM, significantly correlated with AMA_choice_ and all aspects of narrative structure. AMA_choice_ also significantly correlated with ARC, EMA, macro- and total narrative structure, while AMA_deriv_ correlated with all EFL skills, e.g., EMA and ERC, as well as with all aspects of narrative structures. Moreover, we saw significant correlations between HMA_pseudo_ and both AMA tasks. Similar to the Hebrew sample, ARC showed strong correlation with ERC. As with the Hebrew speakers, we saw significant inter-correlations between micro- and macro-structures as well as with Total score among Arabic-speaking children (see Table 4 for all results).

**Table 4:** Arabic speakers: correlations among all measures and English oral narrative scores.

**Variables**	**1**	**2**	**3**	**4**	**5**	**6**	**7**	**8**	**9**	**10**	**11**	**12**
1. E Micro	–											
2. E Macro	0.70^**^	–										
3.Total Nar	0.95^**^	0.88^**^	–									
4. Raven	0.43^**^	0.36^**^	0.44^**^	–								
5.AMA_deriv_	0.28^*^	0.25^*^	0.29^**^	0.14	–							
6.AMA_choi_	0.20	0.23	0.23	0.05	0.25^*^	–						
7. A RC	0.35^**^	0.33^**^	0.37^**^	0.40^*^	0.25^*^	0.41^**^	–					
8. HMA_real_	0.26^*^	0.28^**^	0.30^**^	0.22^*^	0.09	0.22	0.40^**^	–				
9.HMA_pseudo_	0.31^**^	0.34^**^	0.36^**^	0.10	0.22^*^	0.29^**^	0.42^**^	0.46^**^	–			
10. E WR	0.24^*^	0.31^**^	0.29^**^	0.201	0.09	0.39^**^	0.32^**^	0.21	0.23^*^	–		
11. EMA	0.49^**^	0.52^**^	0.55^**^	0.26^*^	0.24^*^	0.29^**^	0.09	0.21	0.44^**^	0.38^**^	–	
12. E RC	0.46^**^	0.38^**^	0.46^**^	0.34^**^	0.26^*^	0.16	0.42^**^	0.33^**^	0.37^**^	0.24^*^	0.63^**^	–

*AMA_choice_, Arabic morphological root pattern awareness task; AMA_deriv_, Arabic morphological word derivation task; HMA_real_, Hebrew morphological real word derivation task; HMA_pseudo_, Hebrew morphological pseudo word derivation task; RC, reading comprehension; H, Hebrew; E, English; MA, morphological awareness; WR, word repetition*.

* *p < 0.05*.

** *p < 0.01*.

##### Regression Analyses

The Total Narrative score was again our dependent measure, however, in addition to MSA measures, Hebrew L3 tasks were added to the models. The predictor models for EFL narratives among Arabic speakers yielded distinctly different results from the Hebrew models. Model 1 included the same predictors as for Hebrew speakers, e.g., cognitive variables. Arabic linguistic tasks (MA and RC) were added to Model 2. In Model 3 we added the two Hebrew measures of MA, as L3 skills for Arabic speakers. EFL variables of ERC and EMA were entered as Model 4 (see Table 5). The final model was significant and explained 42% of the variance in the total narrative score [*F*_(2,72)_ = 5.25 *p* < 0.001]. Two predictors were found to have a significant association with the dependent variable: Raven (β = 0.29, *p* < 0.01) and EMA (β = 0.33, *p* < 0.01). No significant associations were found for the other variables in the model. The regression model for Arabic is presented in Table 5.

**Table 5:** Hierarchal linear regression analysis for contributors to total narrative score among Arabic speakers.

	**Model 1**			**Model 2**			**Model 3**			**Model 4**		
**Variable**	***B***	***SE B***	**β**	***B***	***SE B***	**β**	***B***	***SE B***	**β**	***B***	***SE B***	**β**
Raven	1.20	0.32	0.38^***^	1.04	0.32	0.33^***^	1.04	0.31	0.33^***^	0.92	0.31	0.29^***^
English word repetition	0.37	0.17	0.22^**^	0.25	0.18	0.14	0.21	0.18	0.13	0.07	0.17	0.04
Arabic morphological word derivation				0.45	0.27	0.17	0.40	0.27	0.15	0.31	0.26	0.11
Arabic morphological choice				0.03	0.10	0.04	0.02	0.10	0.02	0.01	0.09	0.02
Arabic reading comprehension				0.12	0.07	0.19	0.07	0.07	0.12	0.02	0.07	0.03
Hebrew morphological real word derivation							0.05	0.12	0.04	−0.01	0.11	−0.01
Hebrew morphological pseudo word derivation							0.12	0.07	0.198	0.07	0.07	0.12
English morphological awareness										0.16	0.06	0.33^**^
English reading comprehension										0.03	0.06	0.06
*R^2^*		0.22			0.30			0.34		0.42		
*R^2^* change		0.22			0.08			0.04		0.09		
*F* for change in *R^2^*				*F*_(3,76)_ = 3.02 *p* = 0.04			*F*_(2,74)_ = 2.03 *p* = 0.14			*F*_(2,72)_ = 5.25 *p* = 0.01		

** *p is significant at 0.01*;

*** *p is significant at 0.001*.

### Research Question Four

**Figure 1:**
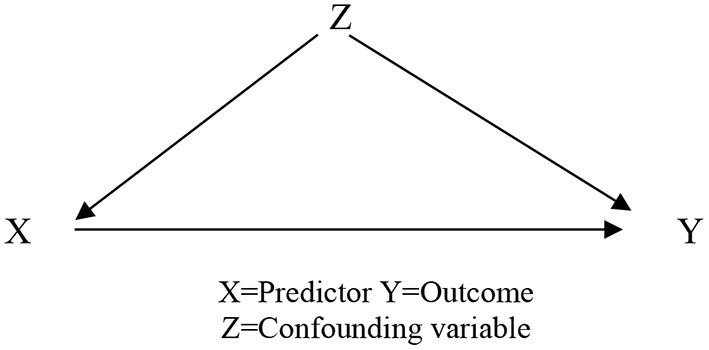
Model for identifying the confounder variable.

Our final research question addressed the possible role of L1/L2/L3 skills in the production of EFL narratives. In essence, we were looking for a specific L1/L2/L3 skill that may give additional support to the quality of EFL oral narratives, based on the MacKinnon et al. (2000) suggestion that after establishing the relationship between predictor and outcome variables it is common to identify a third variable (e.g., specific skill) that may play a role in that relationship. That skill (or variable) should have an influence on both, the predictor variable and outcome variable. This third variable should relate to both factors and possibly enhance the relationship between them. If we identify the predictor variable as X and the outcome variable as Y, variable Z (confounding variable) should have an effect on both, X and Y, therefore signifying cross linguistic transfer from L1(L2/L3) to EFL. These relationships are represented in Figure 1. In what follows, we present the results for the regression analysis for confounding factors.

**Figure 2:**
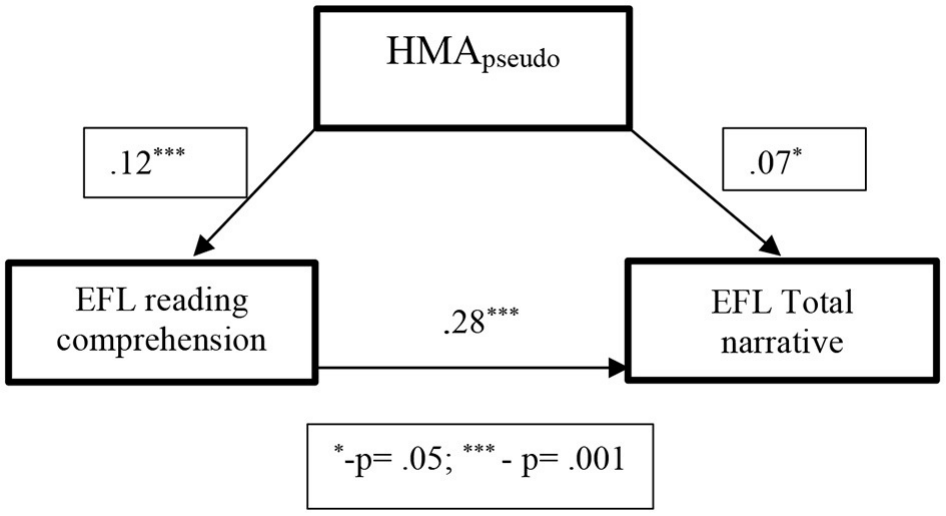
The confounding effect of HMA_pseudo_ on EFL reading comprehension and EFL total narrative score among Hebrew speakers, based on the *R*^2^-values of the regression analysis for confounding factors. *p = 0.05, ***p = 0.001.

Among the Hebrew speakers, this regression analysis indicated that ERC was the strongest EFL predictor for total oral narrative scores [*R*^2^ = *0.28, F*_(1,84)_ = *31.026, p* ≤ *0.001*]. Contrary to the possible assumption that HRC may have some influence on the predictor and outcome variables based on the significant relationship between these variables found in the correlational analysis, this was not the case for Hebrew speakers. While there was strong correlation between ERC and HRC, no correlation was found between the Total Narrative score and HRC, therefore, violating the assumption that the confounding variable should have an influence on both the predictor and the outcome variable (MacKinnon et al., 2000). However, HMA_pseudo_ was found to relate to both, ERC and Total Narrative score. The fact that HMA_pseudo_ can be the influential variable in EFL Total Narrative score is supported by numerous studies, that suggest strong relationship between MA and RC and MA and oral language development (Tomasello, 2005; Verhoeven, 2017). The choice of HMA_pseudo_ as possible variable that may enhance the strength of the relationship between EFL skills and EFL narratives was dictated by the strong metalinguistic component of this particular task: manipulation of pseudo words implies strong knowledge of the underlying mechanism in creating appropriate morphosyntactic elements (Shahar Yames et al., 2018). Therefore, based on the assumption of the confounding hypothesis regarding the effect of a third variable in enhancing the relationship between the predictor and outcome variable, we wanted to see if HMA_pseudo_ would have an effect on both, the ERC and Total narrative score. Indeed, the effect of HMA_pseudo_ on ERC was significant, suggesting that it would increase the ERC (predictor variable) score by 12% [*R*^2^ = *0.122, F*_(1,84)_ = *11.48, p* < *0.001*]. The effect of the HMA_pseudo_ on Total Narrative (outcome variable) score was also significant and suggested the increase on the Total Narrative score of 7% [*R*^2^ = *0.073, F*_(1,84)_ = *6.601, p* < *0.01*). Thus, we could say that HMA_pseudo_ had a significant effect on both, the predictor and the outcome variables, which was confirmed by bootstrapping procedures showing a confidence interval range from 0.01 to 0.13 and Sobel test (*z* = *2.08, p* = *0.04*) (See Figure 2).

**Figure 3:**
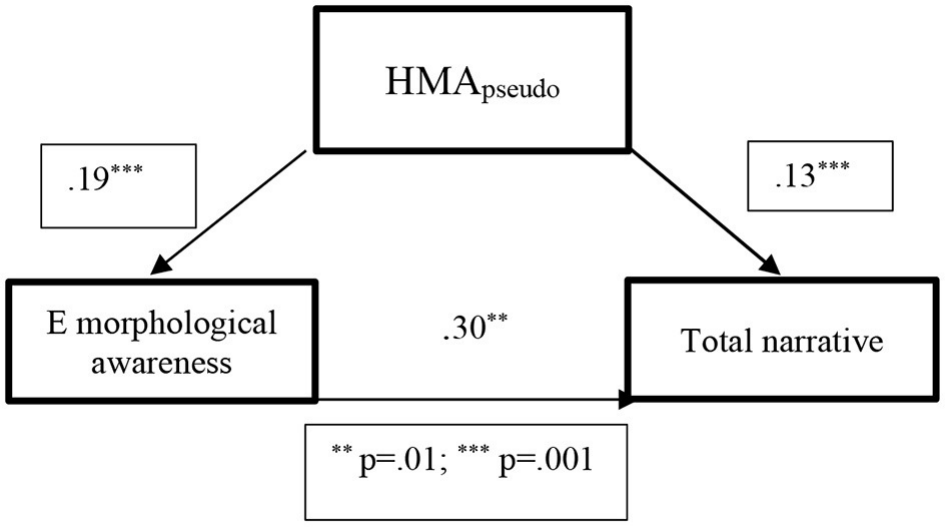
The confounding effect of HMA_pseudo_ on EFL morphological awareness and EFL total narrative score among Arabic speakers, based on the *R*^2^-values of the regression analysis for confounding factors. **p = 0.01, ***p = 0.001.

Among the Arabic speakers, the regression analysis for confounding factors indicated that English MA was the strongest predictor for the Total oral narrative scores [*R*^2^ = *0.302, F*_(1,82)_ = *35.445, p* ≤ *0.001*], accounting for 30% of the variance. Again, our assumption that AMA may support EFL narratives did not yield the expected results. However, among Arabic speakers, AMA_choice_ and HMA_pseudo_ variables showed the strongest and most significant correlations. Moreover, significant correlations among this group were also found between HMA_pseudo_ and EMA, as well as Total Nrrative score. As stated above, the relatively stronger performance of Arabic speakers in the task of HMA_pseudo_, as opposed to HMA_real_ signified their developed metacognitive and metalinguistic skills, based on the exposure to multiple languages (Jessner, 2008). Therefore, we assumed that HMA_pseudo_ may have an effect on EFL Total Narrative score among this group as well. Indeed, HMA_pseudo_ had a significant effect on EMA among Arabic speakers, accounting for 19% of the variance [*R*^2^ = *0.194, F*_(1,82)_ = *19.55, p* < *0.001*]. The effect of HMA_pseudo_ on the Total Narrative score in this group was even stronger than among Hebrew speakers, accounting for almost 13% of the variance [*R*^2^ = *0.129, F*_(1,82)_ = *12.003 p* < *0.001*] and suggesting the cross language influence. Again, the bootstrapping procedures confirmed the significance of the effect, with a confidence interval range from 0.023 to 0.177, as did the Sobel test (*z* = *0.2.27, p* = *0.02*). Therefore, we assumed that HMA_pseudo_, was an influential variable that significantly impacted narrative performance in this group of participants (see Figure 3).

## Discussion

The present study explored cross linguistic cognitive and linguistic Hebrew, MSA, and EFL influences on oral narrative production in EFL among two different Semitic L1 groups: Hebrew and Arabic speakers. We chose to focus our inquiry on oral narrative skills for several reasons: (1) Oral narratives have been identified as a multidimensional measure of language proficiency in any language, as they encompass linguistic skills, i.e., lexical diversity and morphosyntactic knowledge, along with metacognitive abilities and global understanding of narrative structure; (2) Prior research has indicated strong relations between lexical and grammatical knowledge across different languages (Thordardottir et al., 2002), as well as across multiple language speakers, when producing narratives in non-native languages (Marchman et al., 2004; Gutiérrez-Clellen and Simon-Cereijido, 2009; Gagarina et al., 2015; Gagarina, 2016); and (3) Strong cross-linguistic influences have been seen in acquisition and use of morphological and morphosyntactic knowledge among speakers of different languages in their attempts to learn another language (Castilla et al., 2009; Zhang, 2015). Moreover, these influences show bidirectionality (Rezzonico et al., 2016).

It is important to note that while both of Hebrew and Arabic—the two Semitic languages under consideration in the current study—share many typological characteristics, there are also some features that are unique to Arabic resulting from its diglossic nature. Firstly, the distance between the spoken dialects and the MSA form amplifies the overall linguistic morphological complexity. Secondly, the formal acquisition of the MSA form, which coincides with the entry of an Arabic speaking child into school, has been found to hinder the acquisition of literacy and literacy related skills in both the early and later years (Saiegh-Haddad, 2003, 2007; Abdelhadi et al., 2011). Finally, in Israel, the MSA form, essentially an L2 for all Arabic-speaking children, is quickly followed by Hebrew, as an L3 and then EFL, as an L4. In essence, an Arabic speaking child is bilingual before he starts learning Hebrew and then EFL (Eviatar and Ibrahim, 2000), as opposed to a monolingual Hebrew speaking child who acquires EFL as his first additional language (L2). Within this context, it was of particular interest to investigate possible within group relational differences between L1 (Hebrew)/L2 (MSA), and EFL language skills among Arabic and Hebrew speakers for two reasons, (1) while Hebrew and Arabic are both Semitic languages, there are unique linguistic features in Arabic which could lead to different relationships between L2 (MSA) and EFL, as opposed to L1 Hebrew and EFL; (2) despite these differences in linguistic backgrounds, both language groups learn EFL according to the same curricular materials, and are subject to the same national exams.

Within this linguistic setting, our investigation was rooted in specific theoretical assumptions regarding cross-linguistic transfer, and the role played by language typology and L1 proficiency. In line with the *Linguistic Proximity Model* (Westergaard et al., 2017), specific patterns of influence will be determined by the areas of cross language overlap, or structural similarities among the languages of a multilingual learner. Therefore, we assumed that L1 (Hebrew) and L2 (MSA) skills would show shared underlying language learning mechanisms, based on typological proximity. However, we also acknowledged the potential role of the ambient language proficiency in the acquisition of additional languages, as postulated by the *Interdependence Hypothesis* (Cummins, 1979). In the present study, we were interested in exploring if these hypotheses are relevant when considering EFL oral language skills, as measured by the production of oral narratives, in response to a static stimulus. Of specific interest were the issues of cross linguistic influences, as well as identifying modulating and mediating roles of specific EFL and native language skills in producing oral narratives in EFL. Addressing these issues would shed the light on the underlying mechanisms for CLIs between typologically distanced languages.

### The Influence of Cognitive and Native Language Skills Among Hebrew and Arabic Speakers on EFL Oral Narratives Production

Our first and second research questions addressed the influence of cognitive skills, Hebrew and MSA, as well as Hebrew as L3 for Arabic speakers, on all aspects of EFL narrative production. Our hypotheses stated that we should see cross linguistic influences of native language skills on EFL narratives based on levels of language proficiency, as postulated by the *Interdependence Hypothesis* (Cummins, 1979, 2014). Absence of the observed transfers could be attributed to the tenet of the *Linguistic Proximity Model* (Westergaard et al., 2017), suggesting that based on the typological distances between language non-facilitative influences may occur when the learner does not have a solid grasp of particular linguistic input in the target language. Based on these theoretical assumptions, we first looked at the overall psycholinguistic profiles of both linguistic groups in our study, beginning with the Hebrew speaking sample.

As seen in the descriptive statistics, Hebrew speakers showed high scores on measures of L1 proficiency, with higher scores for morphological awareness of real over pseudo words. In EFL, scores on morphological awareness (EMA) were at a moderate high level of proficiency. Scores for reading comprehension (ERC) were the lowest of the English linguistic scores, possibly indicating that despite strong knowledge of individual words, these children still lack the ability to recruit their vocabulary skills in order to comprehend language in context (Masrai, 2019). The scores for EWR as a measure of PM, were close to ceiling. Of interest here is the suggestion proposed by Kaushanskaya et al. (2011) that bilingual individuals may rely on PM for vocabulary retrieval in L2, therefore boosting reading comprehension as well as oral narratives. Furthermore, the fact that the PM measure (EWR) correlated with Hebrew morphology tasks supports the notion that PM is important not only for vocabulary building, but also for the acquisition of morphology independent of native language (Williams and Lovatt, 2003).

Our subsequent analyses, related to our first research question, aimed to identify the specific skills that show cross linguistic influences from L1 to EFL, and to see how these skills support oral language narrative production in EFL. Of interest was the finding that whereas Hebrew morphological awareness tasks (HMA) (real and pseudo words), as part of within language associations, did not show any correlations with Hebrew reading comprehension (HRC), both HMA tasks significantly correlated with EFL reading comprehension. It may be inferred, then, that strong correlations between HMA and ERC suggest that skills in this area of L1 linguistic knowledge are very important for understanding the syntactic structure of L2 (EFL in this instance) in order to fully comprehend written text (Chen and Schwartz, 2018). This correlation exemplifies a cross-linguistic influence, and provides additional support to previous research, which indicated that morphological awareness could transfer from L1 to L2 even among typologically distant languages (Geva, 2006; Yip and Matthews, 2007; Pasquarella et al., 2011). Since HMA also showed strong association with EFL micro-, macro- and total score elements of EFL narratives, we can assume that L1 morphological awareness, as part of morphosyntactic knowledge (James et al., 2021) is a strong component of EFL oral production. Not surprisingly, the EWR as a measure of PM, showed strong associations with both HMA tasks, as well as all EFL narrative structures scores. This particular association provides strong support for the notion that PM is an important underlying cognitive/linguistic skill required for language acquisition. This relationship is seen not only across different L1 languages, but also in acquiring foreign languages as well (Service and Konohen, 1995; Masoura and Gathercole, 2005; Verhagen and Leseman, 2016). Moreover, the regression analysis highlighted the contribution of PM to the production of EFL narratives, solidifying our assumption that PM is important for acquisition of EFL oral language skills. Since Hebrew speakers showed high language proficiency in their ambient language, it supports the main tenet of *Interdependence Hypothesis* (Cummins, 1979, 2014). And yet, despite strong associations between the HMA tasks and all elements of EFL narratives, none of the Hebrew linguistic tasks added to the quality of EFL narratives beyond the contribution of EWR as a measure of PM.

Our second research question addressed the relationship between MSA (L2), L3 (Hebrew, for Arabic speakers), and EFL narratives among the Arabic speakers in the current study. It implied that there may be unique allocation of cognitive and linguistic resources among multilingual Arabic speakers. Our results indicated that our hypothesis was correct, as the results showed different within- and across-languages patterns of associations between tasks, as well as different cognitive and linguistic contributors to oral EFL narrative skills from those of the Hebrew speakers.

As a group, Arabic speakers showed average native language proficiency (according to existing norms, Asadi et al., 2015) and EFL proficiency within the average range, as measured by EMA and ERC scores. While the EFL tasks were not standardized as they were designed for the present study, achieving a result within the 33^rd^-50^th^ percentile can be considered within average range^3^. This was not surprising given the diglossic nature of Arabic and the notion that the existence of two forms of Arabic (the spoken dialect and MSA form) may interfere in acquisitional processes (Saiegh-Haddad, 2003, 2007). Moreover, the Arabic speakers in this study also had to contend with Hebrew as their third spoken and second written language. However, Arabic speakers showed strong results on EWR task as a measure of PM, as could be expected from individuals who are exposed to multiple phonological systems (Bialystok et al., 2003). This group also showed different pattern of results on the HMA tasks. In contrast to the Hebrew speakers, the Arabic speakers scored higher on the pseudo word derivation task than on the real word derivation task. This could be due to the fact that deriving a real word is more reliant on lexical knowledge, whereas deriving a pseudo word is more dependent on the underlying morpho-syntactic knowledge and internalization of rules and patterns (Williams and Lovatt, 2003). Thus, their scores would indicate that while their word level knowledge in Hebrew may be lower, their understanding of how words are formed may be heightened as a result of their experiences with multiple languages (Kuo and Anderson, 2006). Nevertheless, we also saw the same inter-language relationships among Arabic speakers in MSA measures, general cognitive skills and EWR, as a measure of PM, as were found among Hebrew speakers again indicating the important role of PM in language acquisition across languages.

Correlational analysis showed very strong associations between Raven (general cognition) and ARC, and every EFL measure, which was not the case for Hebrew speakers. Therefore, we can postulate that general cognitive abilities play an important role in language acquisition among this group of children. The reliance on general cognition for linguistic tasks has been postulated to be a driving force in language development (Clark, 2004; Tomasello, 2005). Tomasello (2005) specifically identified “pattern-finding…” as a “…cognitive skill involved in the abstraction process” (p. 193), which leads to integration of perceptual information into children's linguistic repertoire (Clark, 2004). However, Raven also showed strong correlations with ARC and HMA for real words (HMA_real_). One possible explanation could suggest that Arabic speakers were relying on general cognitive resources to retrieve vocabulary items for reading comprehension in their native language, as well as recalling Hebrew vocabulary words to apply derivational process by association, rather than relying only on PM, although previous research found no connection between Raven and vocabulary (Ordónez et al., 2002). On the other hand, we also saw strong associations between PM and AMA and ARC, as well as with all aspects of narrative structure. This finding is not surprising since PM is strongly related to vocabulary development (Gathercole et al., 1999), and has been found to strongly associate with both MA and RC, a skill which is comprised of decoding abilities, lexical knowledge, as well as knowledge and understanding of morphosyntactic structures (Hipfner-Boucher et al., 2015). As Arabic morphology is very rich, PM provides the basis in the development of all of the abovementioned skills, and in turn may support the acquisition of MA in additional languages (Verhagen and Leseman, 2016). Indeed, there were significant correlations between HMA and AMA, as an example of cross-linguistic transfer between two typologically close languages. Additionally, there was a cross-linguistic transfer in MA between Arabic and EFL, a typologically distant language. These results could be interpreted in relation to the *Interdependence Hypothesis*. As PM was one of the strongest skills exhibited by Arabic speakers, we may postulate that the exposure to more than one phonological system has strengthened PM skills in this group. This conclusion gains support from numerous studies showing that bilingual children have stronger PM (Bialystok et al., 2003; Parra et al., 2011; Zaretsky, 2018).

### The Effect of EFL Language Skills in EFL Oral Narrative Production

Our assumptions from the onset of the study were that EFL skills will directly contribute to the EFL oral narrative production. The question was: will there be differences in these contributions among Hebrew and Arabic speakers. To answer this question, we conducted an additional regression analysis for both groups. Indeed, there were between group differences in how and which EFL skills supported oral language narratives. Among Hebrew speakers ERC made the largest contribution to the Total narrative score (combination of micro- and macro-structures). This contribution is not unexpected, as RC involves many elements that comprise narratives, as well as being a combination of lexical and morphosyntactic knowledge on its own. Moreover, Hebrew speakers showed above average scores on ERC (while there are no standard norms for this task since it was designed for this study, achieving a score between 60^th^ and 75^th^ percentile can be considered above average. See footnote 3 above), as well as high average score on HRC. This result is in line with the *Interdependence Hypothesis*, which postulates that L1 proficiency can support L2 skills. The fact that PM also supported EFL narratives in this group, was an additional conformation that this cognitive/linguistic skill is intimately involved in every aspect of language acquisition, from early acquisition of vocabulary (Gathercole et al., 1999) to supporting MA and metalinguistic skills (Kupersmitt et al., 2014; Boerma et al., 2016), as well as being an important skill in the acquisition of narrative abilities and promoting grammatical competence in later stages of L2 acquisition (O'Brien et al., 2006).

As we hypothesized, Arabic speakers relied on different EFL skills to support EFL oral narratives. The multilingual Arabic speakers were strong on the tasks that measured AM. Moreover, based on their HMA performance, specifically on the HMA pseudo word task, we can infer that they have better understanding of morphological procedures, above and beyond specific lexical representation at the word level. This finding is a direct conformation of the proposal presented by Shahar Yames et al. (2018), that suggested a separation of lexical and morphological knowledge among learners of additional language, who show much better performance on morphological tasks that are not dependent on extensive lexical knowledge, as measured in their case by pseudo word tasks. As further evidence of strong morphological awareness skills, the Arabic speakers performed within average range on EMA task (see footnote 3). Thus, it was no surprise to see that EMA was the largest contributor to the EFL Total narrative score. This particular finding was of importance as it provided additional support to previous research findings that MA is essential for lexical development (Zhang, 2015), which in turn impacts oral narrative production. Moreover, the correlations between MA tasks in Arabic, Hebrew and EFL support the idea that cross-linguistic influences can be traced between languages that belong to the same typological group, such as Hebrew and Arabic, as well as languages that are typologically distant (Schwartz et al., 2016; Asli-Badarneh and Leikin, 2019), and further highlights the potential for cross linguistic influences across all the languages in the linguistic repertoire for multilinguals (Cenoz, 2013).

These results also highlighted skills that can be considered as contributors which serve to increase the level of oral language production among Hebrew and Arabic speaking children. It has been empirically shown that bilinguals and multilinguals are influenced by one of their languages in activating the processing of another (Dijkstra, 2005), and this activation is seen for different types of information, including syntactic structures (Macizo et al., 2010). So, it is possible to postulate that ERC is the important influencer for EFL oral narratives for Hebrew speakers, i.e., the stronger the ERC scores, the better the EFL narratives, especially since RC and narratives rely on similar component skills. Further, ERC scores for this group were very strong. However, there was a different influence of EFL narrative scores for Arabic speakers, namely EMA. Influence of MA on narratives skills are well-documented, therefore there is no surprise that there should be an influence of EMA on EFL narratives, particularly in this group. This relationship in this case may be explained by the fact that the Arabic speakers may have heightened morphological awareness skills as part of their cross linguistic metacognitive repertoire (Bialystok et al., 2003; Hirosh and Degani, 2018), resulting from their prior experiences with two Arabic morphological systems (spoken dialect and MSA form), as well as Hebrew MA. This could explain how increases in EMA may reflect an increase in the EFL Total narrative score among this population.

### L1(Hebrew)/L2 (Modern Standard Arabic)/L3 (Hebrew for Arabic Speakers): The Skills That Play an Influential Role in the Relationship Between EFL Skills and EFL Oral Narratives

Our last research question explored the possibility that there may be a specific L1/L2/L3 skill, that could play an influencing role in enhancing the relationship between the predictor and outcome variables. We chose the confounding hypothesis as an explanatory framework in order to exlore this possibility. The confounding hypothesis proposes a relationship whereby an additional variable should be related to the factors of interest (predictor and outcome). Namely, it should be correlated with predictor and related to the outcome. Moreover, this particular hypothesis would strongly support the tenets of *Interdependence Hypothesis*.

As seen in the strength of the EFL skills as predictors for EFL narratives, ERC was the predictor variable for Hebrew speakers. The correlational analysis suggested that HMA_pseudo_ (as the third variable) strongly correlated with ERC, our independent variable (IV, or predictor), therefore fulfilling the first assumption of the confounding hypothesis, namely that the confounding variable and IV should correlate. At the same time, HMA_pseudo_ was related to the EFL Total Narrative score, i.e., it increased the quality of the narrative, fulfilling the second assumption of the confounding hypothesis. Moreover, this explanation aligns with the tenet of the *Interdependence Hypothesis*, in suggesting that strong L1 skills will support L2 skills, indicating a CLI.

Among Arabic speakers, EMA was the predictor variable for the Total narrative score, as evidence of stronger metalinguistic knowledge in this multilingual group as opposed to weaker specific linguistic knowledge. However, AMA was not found to explain the relationship between the predictor (EMA) and the outcome (EFL Total narrative score), e.g., it was not a factor in enhancing the outcome. On the other hand, the strong performance of Arabic speakers on HMA_pseudo_ task also suggested the possibility that HMA may be the 3^rd^ variable that would explain the relationship between IV (EMA) and DV (EFL Total narrative). Our analysis indicated that this was the case for Arabic speakers as well: HMA_pseudo_ was a confounding variable that explained the increase in EFL oral language performance among Arabic speakers. This finding is important because it expands the significance of previous data regarding the role of morphological awareness as L1/L2 linguistic skills (Zhang, 2015), to include additional languages. Moreover, this particular finding provided strong support for previous research findings that MA is essential for vocabulary development (Zhang, 2015), and suggested that it can play a strong role in acquisitional processes.

The findings for both language groups are also in line with Hirosh and Degani (2018) proposition that multilinguals may activate both direct routes and indirect routes to additional language learning, where direct routes include transfer of linguistic skills and knowledge, while indirect routes represent cognitive factors such as metalinguistic awareness and working memory, which was the case among our participants. The present findings gain support from recent research which indicates the importance of crosslinguistic transfer of skills, particularly MA, from L1 to L2 (*forward transfer*) (Jarvis and Pavlenko, 2008; Kim and Piper, 2019). Moreover, we found that a specific MA skill in one language may enhance narrative production among two groups of speakers of different Semitic languages. This skill was HMA_pseudo_, which clearly influenced the EFL Total narrative scores for both linguistic groups, despite typological distance of the languages. It is possible then to postulate that the assumptions of *Linguistic Proximity Model* (Westergaard et al., 2017) that crosslinguistic transfer between typologically distant languages will be determined by the areas of cross language overlap may not necessarily account for the application of all cognitive and linguistic resources among multilingual speakers, since our findings indicated transfer among typologically different languages. Although we did not observe bidirectional transfer of specific skills, the finding that Hebrew MA support EFL narratives not only for native Hebrew speakers but also for Arabic speakers, increases our understanding of the role of CLIs in oral language production.

## Conclusions

The present study provided important insights regarding the cognitive and linguistic skills contributing to oral narrative production in EFL among speakers of Semitic languages: Arabic and Hebrew. In line with the theoretical framework suggested by the *Interdependence Hypothesis* (Cummins, 1979, 1991), we saw cross-linguistic transfer of both cognitive and linguistic skills between Arabic and Hebrew, typologically close languages, as well as cross-linguistic transfer, particularly in morphological awareness, in typologically distant languages (Arabic, Hebrew, and English), although the specific psycholinguistic cross linguistic profiles of each language group were unique. Nonetheless, Hebrew morphological derivation of pseudo words was found to be a confounding variable for total narrative skills in both language groups, thereby adding support to previous findings that MA is an essential skill required for native language acquisition, as well as for acquisition of an additional language (Kuo and Anderson, 2006).

The findings of this study could also have pedagogical repercussions especially in light of the fact that in today's world many pupils study in languages that they do not speak at home (Nag et al., 2019) and specifically in Israel, where pupils from multiple language backgrounds all study English according to the same curriculum. In line with the *Linguistic Proximity Model* (Westergaard et al., 2017), our analyses suggested that even typologically related linguistic groups may exhibit different allocations of cognitive and linguistic resources to achieve L1 and L2 and EFL proficiency. Thus, if pupils from different L1 backgrounds are expected to study according to the same curricular materials and be tested according to the same standards, it is possible that certain implementational modifications in the study programs should be made based on the areas of linguistic overlap and the breadth of the linguistic repertoires of the languages of the learners. This could be as simple as acknowledging fine-grained similarities and differences between languages in a direct manner during the teaching process. Moreover, it is possible that for multilingual learners who have not reached a sufficient level of proficiency to support cross linguistic transfer across languages, it may be prudent to allocate additional hours for extended practice, specifically in situations where English is the third or fourth language of the learners. These implications are particularly important since previous research has highlighted the need for specific and targeted intervention in order to aid EFL learners in acquiring necessary proficiency in English across oral and written modalities (Kahn-Horwiz, 2020).

## Data Availability Statement

The raw data supporting the conclusions of this article will be made available by the authors, without undue reservation.

## Ethics Statement

The studies involving human participants were reviewed and approved by Chief Scientist of the State of Israel. Written informed consent to participate in this study was provided by the participants' legal guardian/next of kin.

## Author Contributions

All authors listed have made a substantial, direct and intellectual contribution to the work, and approved it for publication.

## Conflict of Interest

The authors declare that the research was conducted in the absence of any commercial or financial relationships that could be construed as a potential conflict of interest.

## Publisher's Note

All claims expressed in this article are solely those of the authors and do not necessarily represent those of their affiliated organizations, or those of the publisher, the editors and the reviewers. Any product that may be evaluated in this article, or claim that may be made by its manufacturer, is not guaranteed or endorsed by the publisher.

## Footnotes

^1^ https://meyda.education.gov.il/files/Mazkirut_Pedagogit/English/curriculum2020Elementary.pdf

^2^In this study, we are comparing the mean performance of each group and not individuals. Since performance by each group on the native language tasks was at least within the high average range on all native language tasks, we do not consider the differences in SES to be an obstacle.

^3^On a teacher made or non-standardized test, as was the case with our experimental measures, raw scores represent the number of correct responses out of x number of questions. In our case percent correct was used to judge the child's ability on specific task. In line with what is generally accepted in psychological/psycholinguistic testing, any number above 50–60 percentile will be considered above average. Any number above 75 percentile will be high average. Subsequently, any number between 35 and 50 percentile will be within average range, below 35 percentile below average and below 16 percentile low score (Rumsey, 2011).
