# Supplementary material for: Cognitive and Linguistic Skills Associated With Cross-Linguistic Transfer in the Production of Oral Narratives in English as a Foreign Language by Arabic- and Hebrew-Speaking Children: Finding Common Denominators
Source: Front Psychol. 2021 Aug 9;12:664152. doi: 10.3389/fpsyg.2021.664152 (PMC8381353; doi:10.3389/fpsyg.2021.664152)
Supplement: Supplementary file 1 [file Data_Sheet_1.pdf]

Appendix 1

Narrative Assessment Protocol for Micro- and Macro-structures (after Justice et al., 2010 and Heilmann et al., 2010).

Microstructure

Index

Complete Sentences (CS)	Examples	Frequency						
		Plurals	Aux/verb	Past	3^{d} pres	Copula	WOrder	Total
This index included: appropriate inflectional morphology (plurals, tenses, etc.) and correct word order	*Jack has a cookie*	0	0	0	1	1	1	3
	Mom is wash the….	0	0 (-ing)	0	0	0	1	1

Conjunction Cohesion (CC)	Examples	Score	Total Elements
Measure of sentences complexity, e.g., use of clausal structures, as well as appropriate inflectional morphology and word order.	*The boy can't reach the cookie jar, so he decided to stand on the chair.*	1	5 (has all required elements)
	The mom wash and she see in the window.	0	0 (Missing core elements)
**Total Score: sum of the CS and CC**	**Possible score - 36 points**		

Macrostructure[1]

The *"Cookie Theft"* story was divided into three (3) episodes: 1) Mother (washing dishes, holding plate, drying dishes, looking out of the window, etc.); 2) boy/girl (reaching for cookies, giving cookies, eating cookies, climbing chair, etc.); and 3) water overflowing from the sink (water on the floor, wet floor, mother/children don't see it, etc.). To account for completeness of the story, we used the following five (5) indices, which were scored on a 0-3 scale, where 0 signified non-observed ability and 3 indicates all the elements are present, resulting in 15 points total.

[1] Note: The Total Narrative Score was a sum of micro- and macro structures with the possible total score of 51.

Scoring system

Indices	1 point	2 points	3 points
1. Topic Maintenance: Does the story contain all three required episodes?	Only one episode is mentioned (any episode)	Two episodes (any of the 3)	All three episodes are mentioned
2. Event Sequencing (Cause and effect): Does information follow the chronological order, e.g. one action is linked to another (water open – flood on the floor; boy on a chair and falling; mother doesn't see the water flood/children taking cookies)?	Only one episode has sequential order	Events are presented in chronological order within any two episodes	All episodes follow chronological order
3. Information: Does the child give information about the setting (kitchen, house…), action (washing, giving, reaching, etc…), provides supporting details (clothes protagonists wear, hair, size, surroundings, e.g., trees, etc.)?	Only one piece of information is present (no setting, but maybe action, in all episodes)	Two pieces of information are present (maybe setting and another detail (action)	Three informational components are present
4. Referencing: Does the child use pronouns correctly (mother/girl-she, boy – he, children-they) with antecedence (listener can identify which character the pronoun refers to):	Nonspecific pronoun without indicating who is being talked about (i.e., he, she, him, her, they, them)	1 specified pronoun (mother and then 'she' in the sentences that describe what mother is doing; the same is for any child in the picture)	Consistent and appropriate referencing: pronouns always follow specific person
5. Character ID: Does the child identify the protagonists (mother, boy, girl, children, etc.) and maybe give them proper names?	Identifies 1 or 2 people in the story (either, boy, girl, mother)	Identifies all people in the story	Gives the protagonist proper names (Anita, David, etc., or identifies children as brother and sister)
