# Supplementary material for: Cognitive and Linguistic Skills Associated With Cross-Linguistic Transfer in the Production of Oral Narratives in English as a Foreign Language by Arabic- and Hebrew-Speaking Children: Finding Common Denominators
Source: Front Psychol. 2021 Aug 9;12:664152. doi: 10.3389/fpsyg.2021.664152 (PMC8381353; doi:10.3389/fpsyg.2021.664152)
Supplement: Supplementary file 2 [file Data_Sheet_2.pdf]

Appendix 2

Abbreviations used in the paper

A – Arabic

AMA_{choice} – Arabic morphological root pattern awareness task

AMA_{deriv} – Arabic morphological word derivation task

MSA – Modern Standard Arabic

EFL - English as a foreign language

H – Hebrew

HMA_{pseudo} – Hebrew morphological pseudo word derivation task

HMA_{real} – Hebrew morphological real word derivation task

L1 – first language

L2 – second language

E - English

EMA – English morphological awareness

PM – phonological memory

ERC – English reading comprehension

EWR – English word repetition (as a measure of PM)
